# *N*,*N*-Diethyl­anilinium 2,4-dioxo-5-(2,4,6-trinitro­phen­yl)-1,2,3,4-tetra­hydro­pyrimi­din-6-olate

**DOI:** 10.1107/S1600536811049506

**Published:** 2011-11-30

**Authors:** Manickam Buvaneswari, Doraisamyraja Kalaivani

**Affiliations:** a PG and Research Department of Chemistry, Seethalakshmi Ramaswami College, Tiruchirappalli 620 002, Tamil Nadu, India

## Abstract

In the crystal structure of the title mol­ecular salt, C_10_H_16_N^+^·C_10_H_4_N_5_O_9_^−^, the components are linked through a N—H⋯O hydrogen bonds. *R*_2_^2^(8) ring motifs are formed between inversion-related barbiturate residues. Two intra­moleculer N—H⋯O hydrogen bonds are observed in the anion. The dihedral angle between 2,4,6-trinitro­phenyl and barbiturate rings is 53.6 (2)°. The *N*,*N*-diethyl­amine substituent is disordered and was modeled as two geometrically equivalent conformers with occupancies of 0.737 (2) and 0.273 (2).

## Related literature

*N*,*N*-Dialkyl­aniline (aromatic amine) usually forms donor–acceptor adducts with electron-deficient nitro aromatics, see: Radha *et al.* (1987 ▶); Rizk *et al.* (1993 ▶). For similar structures containing the barbiturate anion, see: Buvaneswari & Kalaivani (2011 ▶); Kalaivani & Buvaneswari (2010 ▶); Kalaivani & Malarvizhi (2009 ▶); Kalaivani *et al.* (2008 ▶). 
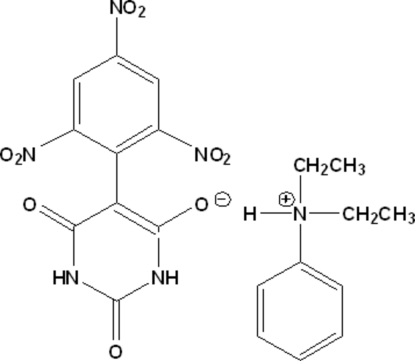


## Experimental

### 

#### Crystal data

C_10_H_16_N^+^·C_10_H_4_N_5_O_9_^−^
*M* *_r_* = 488.42
Monoclinic, 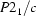

*a* = 17.1903 (7) Å
*b* = 10.3925 (5) Å
*c* = 13.3613 (5) Å
β = 110.272 (2)°
*V* = 2239.14 (16) Å^3^
*Z* = 4
Mo *K*α radiation
μ = 0.12 mm^−1^
*T* = 293 K
0.30 × 0.20 × 0.20 mm

#### Data collection

Bruker Kappa APEXII CCD diffractometer
Absorption correction: multi-scan (*SADABS*; Sheldrick, 2001) ▶ *T*_min_ = 0.923, *T*_max_ = 0.962
3550 measured reflections
3550 independent reflections
2763 reflections with *I* > 2σ(*I*)
θ_max_ = 24.1°

#### Refinement

*R*[*F* ^2^ > 2σ(*F* ^2^)] = 0.038
*wR*(*F* ^2^) = 0.105
*S* = 1.03
3550 reflections
337 parameters
9 restraints
H-atom parameters constrained
Δρ_max_ = 0.20 e Å^−3^
Δρ_min_ = −0.17 e Å^−3^

### 

Data collection: *APEX2* (Bruker, 2004 ▶); cell refinement: *APEX2* and *SAINT* (Bruker, 2004 ▶); data reduction: *SAINT* and *XPREP* (Bruker, 2004 ▶); program(s) used to solve structure: *SHELXS97* (Sheldrick, 2008 ▶); program(s) used to refine structure: *SHELXL97* (Sheldrick, 2008 ▶); molecular graphics: *ORTEP-3* (Farrugia, 1997 ▶) and *Mercury* (Macrae *et al.*, 2008 ▶); software used to prepare material for publication: *SHELXL97*.

## Supplementary Material

Crystal structure: contains datablock(s) global, I. DOI: 10.1107/S1600536811049506/bv2193sup1.cif

Structure factors: contains datablock(s) I. DOI: 10.1107/S1600536811049506/bv2193Isup2.hkl

Supplementary material file. DOI: 10.1107/S1600536811049506/bv2193Isup3.cml

Additional supplementary materials:  crystallographic information; 3D view; checkCIF report

## Figures and Tables

**Table 1:** Hydrogen-bond geometry (Å, °)

*D*—H⋯*A*	*D*—H	H⋯*A*	*D*⋯*A*	*D*—H⋯*A*
N1—H1*A*⋯O1^i^	0.86	2.01	2.8451 (18)	162
N2—H2*A*⋯O3^ii^	0.86	1.98	2.8230 (16)	167
N6*A*—H6*AA*⋯O2	0.91	1.88	2.790 (3)	175
N6*B*—H6*BA*⋯O2	0.91	1.71	2.617 (8)	174

Symmetry codes: (i) 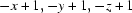; (ii) 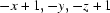.

**Fig. 1.**
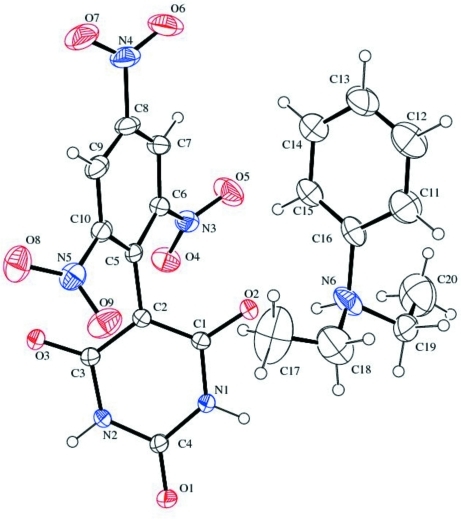
ORTEP view of N,N-diethylanilinium 2,4,6-trinitrophenyl barbiturate showing atom labeling.

**Fig. 2.**
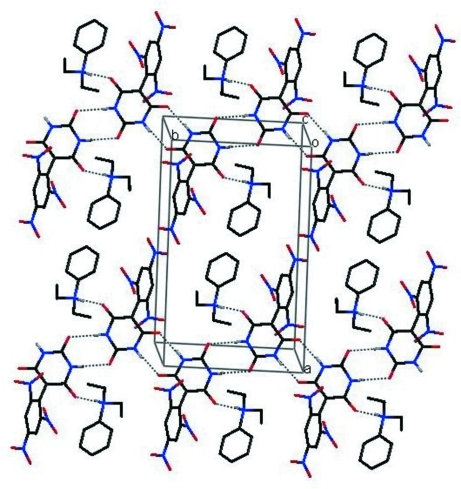
Packing view of N,N-diethylanilinium 2,4,6-trinitrophenyl barbiturate. Hydrogen bonds are shown by dashed lines.

## supplementary crystallographic information

### Comment

*N*,*N*-Dialkylaniline (aromatic amine) usually forms donor-acceptor adducts with electron-deficient nitro aromatics (Radha *et al.*, 1987, Rizk *et al.*, 1993). In the present investigation, it forms a different type of molecular salt (scheme) with the electron-deficient nitro aromatic compound, 1-chloro-2,4,6-trinitrobenzene (picryl chloride) in the presence of barbituric acid. We have already reported molecular salts of a similar type obtained from chlorine containing nitro aromatic compounds, barbituric acid and aliphatic amines (Kalaivani *et al.*, 2008, Kalaivani & Malarvizhi, 2009, Kalaivani & Buvaneswari, 2010, Buvaneswari & Kalaivani, 2011). As noticed in other barbiturates, ring motifs are observed in the crystal structure of the title molecule. The protonated nitrogen atom of *N*,*N*-diethylaniline forms a hydrogen bond with the oxygen atom of the barbiturate anion and this may probably be the driving force for the formation of the title molecular salt.The negative charge on the oxygen atom of olate is delocalized over the nitro groups of the trinitrophenyl moiety and due to this extended conjugation the salt appears bright maroon red in colour.The title molecular salt is obtained with high purity in good yield (90%). Fig.1and 2 represent the *ORTEP* and packing view of the title molecule.

### Experimental

Picryl chloride(1.3.g, 0.005 mol) was dissolved in 15 ml absolute alcohol. Barbituric acid (0.6 g, 0.005 mol)was dissolved in 30 ml of absolute alcohol. These two solutions were mixed and heated to 50°C.To this hot mixture, 4 ml of *N*,*N*-diethylaniline (0.03 mol) was added and shaken well for 3hrs. The crystals obtained were filtered, washed with 50 ml of dry ether and recrystallized from absolute alcohol (yield of pure crystals 90%, m.p.> 573 K). Maroon red block-like single crystals, suitable for X-ray diffraction analysis, were obtained by slow evaporation of an ethanolic solution of the title compound at room temperature.

### Refinement

H atoms bonded to C atoms were placed in their geometrically calculated positions and refined using the riding model, with C–H distances 0.93 - 0.97Å (N–H = 0.86 Å) and *U*_iso_(H) = 1.2 *U*_eq_(C) [*U*_iso_(H) = 1.5 *U*_eq_(CH_3_)]. The N,N-diethylamine substituent is disordered and was modeled as two geometrically equivalent conformers with occupancies of 0.737 (2) and 0.273 (2).

### Crystal data

**Table:**

C_10_H_16_N^+^·C_10_H_4_N_5_O_9_^−^	*F*(000) = 1016
*M**_r_* = 488.42	*D*_x_ = 1.449 Mg m^−^^3^
Monoclinic, *P*2_1_/*c*	Mo *K*α radiation, λ = 0.71073 Å
Hall symbol: -P 2ybc	Cell parameters from 5399 reflections
*a* = 17.1903 (7) Å	θ = 2.4–23.5°
*b* = 10.3925 (5) Å	µ = 0.12 mm^−^^1^
*c* = 13.3613 (5) Å	*T* = 293 K
β = 110.272 (2)°	Block, red
*V* = 2239.14 (16) Å^3^	0.30 × 0.20 × 0.20 mm
*Z* = 4	

### Data collection

**Table:**

Bruker Kappa APEXII CCD diffractometer	3550 independent reflections
Radiation source: fine-focus sealed tube	2763 reflections with *I* > 2σ(*I*)
graphite	*R*_int_ = 0.000
ω and φ scan	θ_max_ = 24.1°, θ_min_ = 2.3°
Absorption correction: multi-scan (*SADABS*; Sheldrick, 2001)	*h* = −19→18
*T*_min_ = 0.923, *T*_max_ = 0.962	*k* = 0→11
3550 measured reflections	*l* = 0→15

### Refinement

**Table:**

Refinement on *F*^2^	Primary atom site location: structure-invariant direct methods
Least-squares matrix: full	Secondary atom site location: difference Fourier map
*R*[*F*^2^ > 2σ(*F*^2^)] = 0.038	Hydrogen site location: inferred from neighbouring sites
*wR*(*F*^2^) = 0.105	H-atom parameters constrained
*S* = 1.03	*w* = 1/[σ^2^(*F*_o_^2^) + (0.0501*P*)^2^ + 0.6573*P*] where *P* = (*F*_o_^2^ + 2*F*_c_^2^)/3
3550 reflections	(Δ/σ)_max_ < 0.001
337 parameters	Δρ_max_ = 0.20 e Å^−^^3^
9 restraints	Δρ_min_ = −0.17 e Å^−^^3^

### Special details

**Table:**

Geometry. All esds (except the esd in the dihedral angle between two l.s. planes) are estimated using the full covariance matrix. The cell esds are taken into account individually in the estimation of esds in distances, angles and torsion angles; correlations between esds in cell parameters are only used when they are defined by crystal symmetry. An approximate (isotropic) treatment of cell esds is used for estimating esds involving l.s. planes.
Refinement. Refinement of F^2^ against ALL reflections. The weighted R-factor wR and goodness of fit S are based on F^2^, conventional R-factors R are based on F, with F set to zero for negative F^2^. The threshold expression of F^2^ > 2sigma(F^2^) is used only for calculating R-factors(gt) etc. and is not relevant to the choice of reflections for refinement. R-factors based on F^2^ are statistically about twice as large as those based on F, and R- factors based on ALL data will be even larger.

### Fractional atomic coordinates and isotropic or equivalent isotropic displacement parameters (Å^2^)

**Table:**

	*x*	*y*	*z*	*U*_iso_*/*U*_eq_	Occ. (<1)
O1	0.55484 (7)	0.34991 (11)	0.49281 (10)	0.0450 (3)	
O2	0.29232 (6)	0.41484 (10)	0.48379 (9)	0.0409 (3)	
O3	0.40988 (7)	0.00160 (10)	0.53210 (10)	0.0440 (3)	
O4	0.21699 (8)	0.20296 (15)	0.31509 (10)	0.0606 (4)	
O5	0.11256 (9)	0.30015 (17)	0.33237 (13)	0.0803 (5)	
O6	0.00042 (9)	0.10375 (17)	0.58155 (14)	0.0813 (5)	
O7	0.07485 (10)	−0.03877 (17)	0.68980 (15)	0.0881 (5)	
O8	0.37537 (10)	−0.03623 (15)	0.75870 (11)	0.0737 (4)	
O9	0.41237 (9)	0.15682 (15)	0.73996 (11)	0.0692 (4)	
N1	0.42299 (7)	0.37873 (12)	0.48839 (10)	0.0361 (3)	
H1A	0.4271	0.4600	0.4792	0.043*	
N2	0.47941 (8)	0.17627 (12)	0.50602 (11)	0.0359 (3)	
H2A	0.5187	0.1261	0.5042	0.043*	
N3	0.17306 (9)	0.23115 (15)	0.36562 (12)	0.0484 (4)	
N4	0.06518 (11)	0.04642 (19)	0.62447 (15)	0.0641 (5)	
N5	0.36317 (10)	0.06897 (16)	0.71637 (11)	0.0505 (4)	
C1	0.34912 (9)	0.33433 (15)	0.49459 (12)	0.0321 (4)	
C2	0.34512 (9)	0.20346 (15)	0.51552 (12)	0.0317 (4)	
C3	0.41044 (9)	0.11993 (15)	0.51931 (12)	0.0332 (4)	
C4	0.48971 (9)	0.30453 (15)	0.49560 (13)	0.0336 (4)	
C5	0.27378 (9)	0.15512 (14)	0.53912 (13)	0.0327 (4)	
C6	0.19142 (9)	0.17548 (16)	0.47270 (13)	0.0376 (4)	
C7	0.12349 (10)	0.14351 (18)	0.49944 (15)	0.0472 (5)	
H7	0.0701	0.1631	0.4542	0.057*	
C8	0.13670 (11)	0.08212 (18)	0.59447 (15)	0.0478 (5)	
C9	0.21488 (11)	0.05304 (17)	0.66264 (14)	0.0465 (4)	
H9	0.2229	0.0089	0.7260	0.056*	
C10	0.28116 (10)	0.09117 (16)	0.63455 (13)	0.0386 (4)	
C11	0.17516 (17)	0.6686 (3)	0.6939 (2)	0.0944 (8)	
H11	0.2048	0.7429	0.7222	0.113*	
C12	0.10144 (18)	0.6418 (3)	0.7087 (2)	0.1087 (10)	
H12	0.0811	0.6995	0.7468	0.130*	
C13	0.05792 (17)	0.5336 (3)	0.6692 (2)	0.0902 (8)	
H13	0.0093	0.5154	0.6822	0.108*	
C14	0.08618 (15)	0.4522 (3)	0.6104 (2)	0.0843 (7)	
H14	0.0561	0.3786	0.5813	0.101*	
C15	0.15923 (13)	0.4777 (2)	0.59356 (18)	0.0665 (6)	
H15	0.1779	0.4218	0.5524	0.080*	
C16	0.20396 (9)	0.58369 (19)	0.63669 (15)	0.0555 (5)	
N6A	0.28140 (9)	0.6169 (2)	0.61581 (18)	0.0508 (7)	0.737 (2)
H6AA	0.2861	0.5546	0.5703	0.061*	0.737 (2)
C17A	0.36242 (16)	0.6089 (3)	0.7110 (2)	0.0598 (8)	0.737 (2)
H17A	0.4083	0.6324	0.6882	0.072*	0.737 (2)
H17B	0.3607	0.6698	0.7652	0.072*	0.737 (2)
C18A	0.3765 (2)	0.4769 (4)	0.7577 (2)	0.0781 (10)	0.737 (2)
H18A	0.4310	0.4720	0.8107	0.117*	0.737 (2)
H18B	0.3718	0.4153	0.7024	0.117*	0.737 (2)
H18C	0.3359	0.4586	0.7901	0.117*	0.737 (2)
C19A	0.2816 (2)	0.7424 (3)	0.5574 (3)	0.0638 (9)	0.737 (2)
H19A	0.2816	0.8141	0.6039	0.077*	0.737 (2)
H19B	0.3320	0.7475	0.5403	0.077*	0.737 (2)
C20A	0.2099 (3)	0.7533 (5)	0.4591 (4)	0.1084 (14)	0.737 (2)
H20A	0.2135	0.8320	0.4233	0.163*	0.737 (2)
H20B	0.1600	0.7534	0.4760	0.163*	0.737 (2)
H20C	0.2091	0.6817	0.4134	0.163*	0.737 (2)
N6B	0.28886 (16)	0.5796 (9)	0.6304 (5)	0.0508 (7)	0.263 (2)
H6BA	0.2935	0.5249	0.5795	0.061*	0.263 (2)
C17B	0.3394 (5)	0.5389 (10)	0.7432 (6)	0.0598 (8)	0.263 (2)
H17C	0.3420	0.6074	0.7936	0.072*	0.263 (2)
H17D	0.3159	0.4628	0.7638	0.072*	0.263 (2)
C18B	0.4219 (5)	0.5117 (11)	0.7381 (7)	0.0781 (10)	0.263 (2)
H18D	0.4552	0.4690	0.8023	0.117*	0.263 (2)
H18E	0.4480	0.5911	0.7307	0.117*	0.263 (2)
H18F	0.4163	0.4575	0.6778	0.117*	0.263 (2)
C19B	0.2941 (7)	0.7145 (10)	0.6051 (8)	0.0638 (9)	0.263 (2)
H19C	0.3516	0.7369	0.6184	0.077*	0.263 (2)
H19D	0.2743	0.7669	0.6514	0.077*	0.263 (2)
C20B	0.2441 (10)	0.7433 (17)	0.4914 (12)	0.1084 (14)	0.263 (2)
H20D	0.2631	0.8222	0.4705	0.163*	0.263 (2)
H20E	0.1867	0.7518	0.4840	0.163*	0.263 (2)
H20F	0.2502	0.6745	0.4468	0.163*	0.263 (2)

### Atomic displacement parameters (Å^2^)

**Table:**

	*U*^11^	*U*^22^	*U*^33^	*U*^12^	*U*^13^	*U*^23^
O1	0.0372 (6)	0.0335 (6)	0.0746 (8)	−0.0036 (5)	0.0323 (6)	−0.0024 (6)
O2	0.0355 (6)	0.0302 (6)	0.0627 (7)	0.0057 (5)	0.0241 (5)	−0.0005 (5)
O3	0.0436 (6)	0.0261 (6)	0.0720 (8)	0.0025 (5)	0.0322 (6)	0.0053 (6)
O4	0.0579 (8)	0.0797 (10)	0.0482 (7)	0.0027 (7)	0.0235 (6)	0.0003 (7)
O5	0.0564 (8)	0.0960 (12)	0.0855 (10)	0.0309 (8)	0.0208 (8)	0.0313 (9)
O6	0.0544 (8)	0.0975 (12)	0.1116 (12)	−0.0154 (9)	0.0535 (8)	−0.0263 (10)
O7	0.1076 (11)	0.0718 (11)	0.1226 (12)	−0.0225 (9)	0.0878 (10)	−0.0006 (10)
O8	0.0922 (11)	0.0574 (9)	0.0622 (9)	0.0063 (8)	0.0150 (8)	0.0182 (8)
O9	0.0671 (9)	0.0756 (10)	0.0556 (8)	−0.0225 (8)	0.0096 (7)	0.0040 (7)
N1	0.0354 (7)	0.0232 (7)	0.0567 (8)	0.0000 (6)	0.0248 (6)	0.0023 (6)
N2	0.0313 (7)	0.0268 (7)	0.0563 (8)	0.0036 (6)	0.0236 (6)	0.0000 (6)
N3	0.0389 (8)	0.0511 (10)	0.0536 (9)	−0.0006 (7)	0.0138 (7)	0.0033 (8)
N4	0.0679 (11)	0.0595 (11)	0.0883 (12)	−0.0223 (9)	0.0566 (9)	−0.0235 (10)
N5	0.0605 (10)	0.0497 (10)	0.0437 (8)	−0.0040 (8)	0.0213 (7)	0.0023 (8)
C1	0.0316 (8)	0.0305 (9)	0.0387 (8)	−0.0003 (7)	0.0180 (6)	−0.0021 (7)
C2	0.0300 (8)	0.0283 (8)	0.0409 (8)	0.0002 (7)	0.0175 (6)	−0.0010 (7)
C3	0.0328 (8)	0.0292 (9)	0.0415 (8)	−0.0015 (7)	0.0179 (7)	0.0001 (7)
C4	0.0341 (8)	0.0286 (9)	0.0435 (9)	−0.0013 (7)	0.0201 (7)	−0.0019 (7)
C5	0.0363 (8)	0.0230 (8)	0.0451 (9)	−0.0019 (7)	0.0221 (7)	−0.0055 (7)
C6	0.0358 (9)	0.0333 (9)	0.0485 (9)	−0.0016 (7)	0.0208 (7)	−0.0025 (7)
C7	0.0354 (9)	0.0438 (11)	0.0681 (12)	−0.0048 (8)	0.0250 (8)	−0.0088 (9)
C8	0.0490 (10)	0.0422 (11)	0.0682 (11)	−0.0136 (8)	0.0406 (9)	−0.0126 (9)
C9	0.0634 (11)	0.0361 (10)	0.0535 (10)	−0.0092 (9)	0.0371 (9)	−0.0032 (8)
C10	0.0439 (9)	0.0325 (9)	0.0444 (9)	−0.0031 (8)	0.0218 (7)	−0.0027 (7)
C11	0.1104 (17)	0.0874 (18)	0.1162 (19)	−0.0050 (15)	0.0782 (16)	−0.0365 (15)
C12	0.129 (2)	0.108 (2)	0.132 (2)	0.0100 (19)	0.0998 (18)	−0.0271 (18)
C13	0.0945 (16)	0.0876 (19)	0.120 (2)	0.0151 (16)	0.0767 (15)	0.0126 (16)
C14	0.0768 (15)	0.0693 (16)	0.123 (2)	0.0049 (13)	0.0556 (15)	−0.0040 (15)
C15	0.0652 (13)	0.0612 (14)	0.0858 (14)	0.0126 (11)	0.0423 (11)	−0.0077 (12)
C16	0.0614 (12)	0.0582 (13)	0.0528 (11)	0.0163 (10)	0.0274 (9)	−0.0032 (10)
N6A	0.0481 (9)	0.0417 (18)	0.0603 (11)	0.0222 (9)	0.0158 (8)	−0.0099 (11)
C17A	0.0508 (15)	0.066 (2)	0.0615 (16)	−0.0104 (14)	0.0186 (12)	−0.0075 (14)
C18A	0.083 (3)	0.088 (2)	0.0538 (17)	0.006 (2)	0.0123 (16)	0.0138 (16)
C19A	0.0846 (17)	0.0416 (17)	0.089 (3)	0.0056 (15)	0.0606 (19)	0.0075 (17)
C20A	0.116 (4)	0.103 (3)	0.104 (3)	0.038 (3)	0.035 (3)	0.049 (2)
N6B	0.0481 (9)	0.0417 (18)	0.0603 (11)	0.0222 (9)	0.0158 (8)	−0.0099 (11)
C17B	0.0508 (15)	0.066 (2)	0.0615 (16)	−0.0104 (14)	0.0186 (12)	−0.0075 (14)
C18B	0.083 (3)	0.088 (2)	0.0538 (17)	0.006 (2)	0.0123 (16)	0.0138 (16)
C19B	0.0846 (17)	0.0416 (17)	0.089 (3)	0.0056 (15)	0.0606 (19)	0.0075 (17)
C20B	0.116 (4)	0.103 (3)	0.104 (3)	0.038 (3)	0.035 (3)	0.049 (2)

### Geometric parameters (Å, °)

**Table:**

O1—C4	1.2273 (18)	C14—C15	1.376 (3)
O2—C1	1.2556 (18)	C14—H14	0.9300
O3—C3	1.2420 (19)	C15—C16	1.353 (3)
O4—N3	1.2104 (19)	C15—H15	0.9300
O5—N3	1.2137 (19)	C16—N6B	1.4915 (17)
O6—N4	1.217 (2)	C16—N6A	1.4921 (16)
O7—N4	1.214 (2)	N6A—C19A	1.521 (4)
O8—N5	1.215 (2)	N6A—C17A	1.528 (3)
O9—N5	1.210 (2)	N6A—H6AA	0.9100
N1—C4	1.357 (2)	C17A—C18A	1.491 (4)
N1—C1	1.3804 (19)	C17A—H17A	0.9700
N1—H1A	0.8600	C17A—H17B	0.9700
N2—C4	1.358 (2)	C18A—H18A	0.9600
N2—C3	1.388 (2)	C18A—H18B	0.9600
N2—H2A	0.8600	C18A—H18C	0.9600
N3—C6	1.473 (2)	C19A—C20A	1.462 (5)
N4—C8	1.467 (2)	C19A—H19A	0.9700
N5—C10	1.472 (2)	C19A—H19B	0.9700
C1—C2	1.395 (2)	C20A—H20A	0.9600
C2—C3	1.406 (2)	C20A—H20B	0.9600
C2—C5	1.457 (2)	C20A—H20C	0.9600
C5—C6	1.402 (2)	N6B—C19B	1.452 (11)
C5—C10	1.404 (2)	N6B—C17B	1.516 (10)
C6—C7	1.375 (2)	N6B—H6BA	0.9100
C7—C8	1.368 (3)	C17B—C18B	1.471 (11)
C7—H7	0.9300	C17B—H17C	0.9700
C8—C9	1.371 (3)	C17B—H17D	0.9700
C9—C10	1.376 (2)	C18B—H18D	0.9600
C9—H9	0.9300	C18B—H18E	0.9600
C11—C16	1.367 (3)	C18B—H18F	0.9600
C11—C12	1.377 (4)	C19B—C20B	1.493 (13)
C11—H11	0.9300	C19B—H19C	0.9700
C12—C13	1.352 (4)	C19B—H19D	0.9700
C12—H12	0.9300	C20B—H20D	0.9600
C13—C14	1.354 (4)	C20B—H20E	0.9600
C13—H13	0.9300	C20B—H20F	0.9600
			
C4—N1—C1	125.32 (13)	C16—C15—C14	120.2 (2)
C4—N1—H1A	117.3	C16—C15—H15	119.9
C1—N1—H1A	117.3	C14—C15—H15	119.9
C4—N2—C3	125.07 (13)	C15—C16—C11	120.07 (19)
C4—N2—H2A	117.5	C15—C16—N6B	112.1 (4)
C3—N2—H2A	117.5	C11—C16—N6B	126.9 (4)
O4—N3—O5	124.05 (17)	C15—C16—N6A	121.40 (18)
O4—N3—C6	118.80 (14)	C11—C16—N6A	118.3 (2)
O5—N3—C6	117.11 (16)	N6B—C16—N6A	16.7 (3)
O7—N4—O6	124.83 (18)	C16—N6A—C19A	117.10 (19)
O7—N4—C8	117.53 (18)	C16—N6A—C17A	116.53 (19)
O6—N4—C8	117.64 (19)	C19A—N6A—C17A	108.1 (2)
O9—N5—O8	124.43 (16)	C16—N6A—H6AA	104.5
O9—N5—C10	118.58 (15)	C19A—N6A—H6AA	104.5
O8—N5—C10	116.93 (16)	C17A—N6A—H6AA	104.5
O2—C1—N1	117.73 (14)	C18A—C17A—N6A	111.6 (2)
O2—C1—C2	125.62 (14)	C18A—C17A—H17A	109.3
N1—C1—C2	116.63 (13)	N6A—C17A—H17A	109.3
C1—C2—C3	120.99 (14)	C18A—C17A—H17B	109.3
C1—C2—C5	118.89 (13)	N6A—C17A—H17B	109.3
C3—C2—C5	120.04 (14)	H17A—C17A—H17B	108.0
O3—C3—N2	118.88 (14)	C20A—C19A—N6A	112.1 (3)
O3—C3—C2	124.89 (14)	C20A—C19A—H19A	109.2
N2—C3—C2	116.22 (14)	N6A—C19A—H19A	109.2
O1—C4—N1	122.47 (15)	C20A—C19A—H19B	109.2
O1—C4—N2	122.20 (14)	N6A—C19A—H19B	109.2
N1—C4—N2	115.33 (13)	H19A—C19A—H19B	107.9
C6—C5—C10	113.57 (14)	C19B—N6B—C16	97.3 (6)
C6—C5—C2	123.50 (14)	C19B—N6B—C17B	116.1 (7)
C10—C5—C2	122.79 (14)	C16—N6B—C17B	100.7 (4)
C7—C6—C5	124.14 (16)	C19B—N6B—H6BA	113.6
C7—C6—N3	115.60 (14)	C16—N6B—H6BA	113.6
C5—C6—N3	120.24 (14)	C17B—N6B—H6BA	113.6
C8—C7—C6	118.10 (16)	C18B—C17B—N6B	103.7 (6)
C8—C7—H7	120.9	C18B—C17B—H17C	111.0
C6—C7—H7	120.9	N6B—C17B—H17C	111.0
C7—C8—C9	121.94 (16)	C18B—C17B—H17D	111.0
C7—C8—N4	119.10 (17)	N6B—C17B—H17D	111.0
C9—C8—N4	118.97 (18)	H17C—C17B—H17D	109.0
C8—C9—C10	117.99 (17)	C17B—C18B—H18D	109.5
C8—C9—H9	121.0	C17B—C18B—H18E	109.5
C10—C9—H9	121.0	H18D—C18B—H18E	109.5
C9—C10—C5	124.14 (16)	C17B—C18B—H18F	109.5
C9—C10—N5	115.01 (15)	H18D—C18B—H18F	109.5
C5—C10—N5	120.69 (15)	H18E—C18B—H18F	109.5
C16—C11—C12	118.6 (3)	N6B—C19B—C20B	111.7 (10)
C16—C11—H11	120.7	N6B—C19B—H19C	109.3
C12—C11—H11	120.7	C20B—C19B—H19C	109.3
C13—C12—C11	121.6 (2)	N6B—C19B—H19D	109.3
C13—C12—H12	119.2	C20B—C19B—H19D	109.3
C11—C12—H12	119.2	H19C—C19B—H19D	107.9
C12—C13—C14	119.0 (2)	C19B—C20B—H20D	109.5
C12—C13—H13	120.5	C19B—C20B—H20E	109.5
C14—C13—H13	120.5	H20D—C20B—H20E	109.5
C13—C14—C15	120.4 (3)	C19B—C20B—H20F	109.5
C13—C14—H14	119.8	H20D—C20B—H20F	109.5
C15—C14—H14	119.8	H20E—C20B—H20F	109.5
			
C4—N1—C1—O2	−178.27 (14)	C8—C9—C10—N5	173.85 (15)
C4—N1—C1—C2	3.5 (2)	C6—C5—C10—C9	−1.0 (2)
O2—C1—C2—C3	175.83 (15)	C2—C5—C10—C9	174.90 (16)
N1—C1—C2—C3	−6.1 (2)	C6—C5—C10—N5	−176.29 (15)
O2—C1—C2—C5	−7.4 (2)	C2—C5—C10—N5	−0.4 (2)
N1—C1—C2—C5	170.69 (13)	O9—N5—C10—C9	−130.27 (18)
C4—N2—C3—O3	−177.23 (15)	O8—N5—C10—C9	46.9 (2)
C4—N2—C3—C2	3.7 (2)	O9—N5—C10—C5	45.5 (2)
C1—C2—C3—O3	−176.19 (15)	O8—N5—C10—C5	−137.36 (17)
C5—C2—C3—O3	7.0 (2)	C16—C11—C12—C13	0.7 (5)
C1—C2—C3—N2	2.8 (2)	C11—C12—C13—C14	−2.4 (5)
C5—C2—C3—N2	−173.94 (13)	C12—C13—C14—C15	1.6 (4)
C1—N1—C4—O1	−177.69 (15)	C13—C14—C15—C16	0.8 (4)
C1—N1—C4—N2	2.4 (2)	C14—C15—C16—C11	−2.5 (3)
C3—N2—C4—O1	173.89 (15)	C14—C15—C16—N6B	167.2 (3)
C3—N2—C4—N1	−6.2 (2)	C14—C15—C16—N6A	−177.3 (2)
C1—C2—C5—C6	53.8 (2)	C12—C11—C16—C15	1.7 (4)
C3—C2—C5—C6	−129.40 (17)	C12—C11—C16—N6B	−166.3 (4)
C1—C2—C5—C10	−121.69 (17)	C12—C11—C16—N6A	176.6 (2)
C3—C2—C5—C10	55.2 (2)	C15—C16—N6A—C19A	117.0 (3)
C10—C5—C6—C7	3.6 (2)	C11—C16—N6A—C19A	−57.9 (3)
C2—C5—C6—C7	−172.22 (16)	N6B—C16—N6A—C19A	176.7 (13)
C10—C5—C6—N3	−174.67 (14)	C15—C16—N6A—C17A	−112.9 (3)
C2—C5—C6—N3	9.5 (2)	C11—C16—N6A—C17A	72.3 (3)
O4—N3—C6—C7	−142.67 (17)	N6B—C16—N6A—C17A	−53.1 (11)
O5—N3—C6—C7	35.2 (2)	C16—N6A—C17A—C18A	59.0 (3)
O4—N3—C6—C5	35.8 (2)	C19A—N6A—C17A—C18A	−166.7 (3)
O5—N3—C6—C5	−146.41 (17)	C16—N6A—C19A—C20A	−51.4 (4)
C5—C6—C7—C8	−3.4 (3)	C17A—N6A—C19A—C20A	174.7 (3)
N3—C6—C7—C8	174.92 (16)	C15—C16—N6B—C19B	140.7 (5)
C6—C7—C8—C9	0.4 (3)	C11—C16—N6B—C19B	−50.4 (6)
C6—C7—C8—N4	−179.68 (16)	N6A—C16—N6B—C19B	13.4 (10)
O7—N4—C8—C7	158.40 (18)	C15—C16—N6B—C17B	−100.9 (6)
O6—N4—C8—C7	−21.1 (3)	C11—C16—N6B—C17B	68.0 (7)
O7—N4—C8—C9	−21.7 (3)	N6A—C16—N6B—C17B	131.8 (15)
O6—N4—C8—C9	158.82 (18)	C19B—N6B—C17B—C18B	−85.5 (9)
C7—C8—C9—C10	2.0 (3)	C16—N6B—C17B—C18B	170.8 (7)
N4—C8—C9—C10	−177.89 (15)	C16—N6B—C19B—C20B	−75.9 (11)
C8—C9—C10—C5	−1.7 (3)	C17B—N6B—C19B—C20B	178.4 (10)

### Hydrogen-bond geometry (Å, °)

**Table:**

*D*—H···*A*	*D*—H	H···*A*	*D*···*A*	*D*—H···*A*
N1—H1A···O1^i^	0.86	2.01	2.8451 (18)	162.
N2—H2A···O3^ii^	0.86	1.98	2.8230 (16)	167.
N6A—H6AA···O2	0.91	1.88	2.790 (3)	175.
N6B—H6BA···O2	0.91	1.71	2.617 (8)	174.

Symmetry codes: (i) −*x*+1, −*y*+1, −*z*+1; (ii) −*x*+1, −*y*, −*z*+1.
